# Endocytic membrane turnover at the leading edge is driven by a transient interaction between Cdc42 and GRAF1

**DOI:** 10.1242/jcs.174417

**Published:** 2015-11-15

**Authors:** Monika K. Francis, Mikkel R. Holst, Maite Vidal-Quadras, Sara Henriksson, Rachel Santarella-Mellwig, Linda Sandblad, Richard Lundmark

**Affiliations:** 1 Integrative Medical Biology, Umeå University, Umeå 901 87, Sweden; 2 Medical Biochemistry and Biophysics, Umeå University, Umeå 901 87, Sweden; 3 Molecular Biology, Umeå University, Umeå 901 87, Sweden; 4 European Molecular Biology Laboratory, Meyerhofstr. 1, Heidelberg 69 117, Germany

**Keywords:** Clathrin-independent endocytosis, GRAF1, Cdc42, Cell surface, Actin

## Abstract

Changes in cell morphology require coordination of plasma membrane turnover and cytoskeleton dynamics, processes that are regulated by Rho GTPases. Here, we describe how a direct interaction between the Rho GTPase Cdc42 and the GTPase-activating protein (GAP) GRAF1 (also known as ARHGAP26), facilitates rapid cell surface turnover at the leading edge. Both Cdc42 and GRAF1 were required for fluid-phase uptake and regulated the generation of transient GRAF1-coated endocytic carriers, which were distinct from clathrin-coated vesicles. GRAF1 was found to transiently assemble at discrete Cdc42-enriched punctae at the plasma membrane, resulting in a corresponding decrease in the microdomain association of Cdc42. However, Cdc42 captured in its active state was, through a GAP-domain-mediated interaction, localised together with GRAF1 on accumulated internal structures derived from the cell surface. Correlative fluorescence and electron tomography microscopy revealed that these structures were clusters of small membrane carriers with defective endosomal processing. We conclude that a transient interaction between Cdc42 and GRAF1 drives endocytic turnover and controls the transition essential for endosomal maturation of plasma membrane internalised by this mechanism.

## Abstract (precis)

**Summary:** A transient interaction between Cdc42 and GRAF1 drives endocytic turnover at the leading edge, and controls the transition essential for endosomal maturation of plasma membrane internalised by this mechanism.

## INTRODUCTION

Cell surface dynamics are fundamental to a variety of basic biological processes, such as migration, polarisation and division, as well as protein and lipid trafficking. Intimate coupling to an underlying meshwork of cortical actin keeps the plasma membrane under high tension, providing the cell surface with its vital strength to withstand mechanical stress (Gauthier et al., 2012). Consequently, structural alterations of the cell surface can only be achieved through the intricate coordination of membrane remodelling events and cytoskeletal rearrangements (de Curtis and Meldolesi, 2012). Small G-proteins of the Rho GTPase family are known as master regulators of the cytoskeleton and have been shown to greatly influence membrane tension and plasma membrane turnover by regulating endocytic and exocytic events (Jaffe and Hall, 2005; Ridley, 2001). Rho GTPases are peripherally attached to the membrane through lipid modifications and are active in their GTP-loaded state, where they interact with various effector molecules and function as molecular switches through rounds of GTP hydrolysis. The activity, which is stimulated by external cues, is strictly regulated by GTPase-activating proteins (GAPs) and guanine-nucleotide-exchange factors (GEFs), which promote GTP hydrolysis and facilitate loading of GTP, respectively (Jaffe and Hall, 2005). However, the intricate mechanisms that allow for a cyclical nucleotide exchange in small G-proteins to synchronise membrane and cytoskeletal dynamics are still not understood.

The clathrin-independent carrier (CLIC) pathway, a major pinocytic endocytic route in fibroblasts, facilitates polarised and rapid uptake of fluid, bacterial toxins, glycosylphosphatidylinositol (GPI)-linked receptors and receptors involved in cell adhesion (Howes et al., 2010a). CLICs were originally defined by their dependence on the Rho GTPase Cdc42 and tubular morphology, separating them from clathrin-coated vesicles and caveolae (Hansen and Nichols, 2009; Howes et al., 2010b; Mayor and Pagano, 2007). Carriers derived from this pathway have been shown to fuse into GPI-enriched endosomal compartments (GEECs), which subsequently merge with early endosomes or recycling endosomes (Kalia et al., 2006). The formation of CLICs depends on a poorly characterised molecular system, involving the small G-proteins Cdc42 and Arf1, and their regulatory proteins (Chadda et al., 2007; Kumari and Mayor, 2008; Lundmark et al., 2008; Sabharanjak et al., 2002). Although CLICs account for a major portion of the endocytic turnover of the plasma membrane, very little mechanistic detail is available regarding their formation.

It has previously been shown that GRAF1 (also known as ARHGAP26), a GAP active against Cdc42 (Hildebrand et al., 1996; Jelen et al., 2009; Longenecker et al., 2000), is essential for CLIC uptake (Lundmark et al., 2008). GRAF1 is a dimeric multidomain protein composed of Bin, amphiphysin, RVS161/167 (BAR), pleckstrin homology (PH), GAP and Src homology 3 (SH3) domains. The BAR and PH domains can generate and/or stabilise highly curved endocytic membranes from phosphatidylinositol 4,5-bisphosphate (PIP_2_)-enriched regions, whereas the C-terminal GAP and SH3 domains modulates Cdc42 activity and interacts with regulatory proteins, respectively (Doherty et al., 2011a; Hildebrand et al., 1996; Lundmark et al., 2008; Okada et al., 2011). Loss of GRAF1 downregulates endocytosis and affects cellular processes such as cortical actin remodelling, integrin trafficking, adhesion, spreading, migration and fusion, suggesting that this protein is an important regulator of cell surface dynamics (Doherty et al., 2011a,b; Lundmark et al., 2008; Nonnenmacher and Weber, 2011; Simpson et al., 2008; Stergiou et al., 2013).

In this work, we studied the interplay between GRAF1 and Cdc42 to decipher its importance for coordination of membrane and actin dynamics during cell surface turnover at the leading edge. We characterise GRAF1 as a molecular marker of CLICs and demonstrate how this protein, through a direct interaction, regulates Cdc42 activity during endocytosis. This study also defines a temporal and spatial restriction of active Cdc42 as a prerequisite for the internalisation and further trafficking of cell surface components.

## RESULTS

### Correlative light and electron microscopy reveals highly curved membrane carriers decorated by GRAF1 and GTPase-deficient Cdc42

**Fig. 1.**
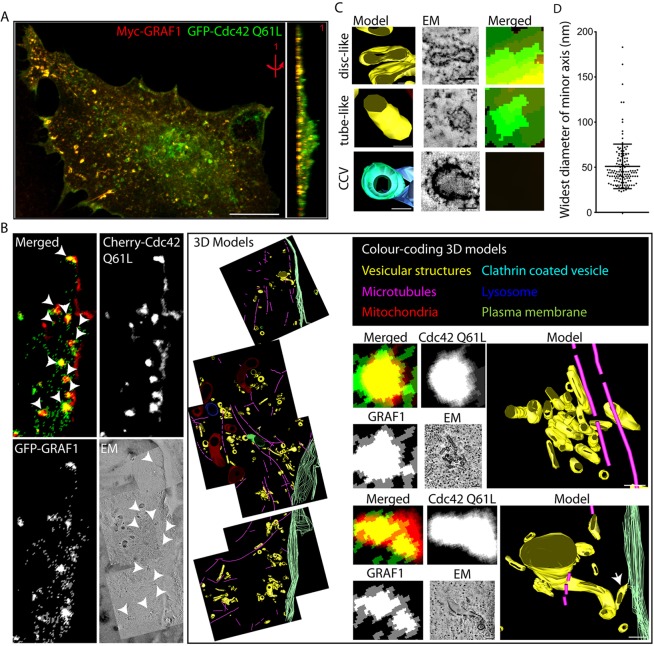
**GRAF1 and GTPase-deficient Cdc42 localise to surface-detached pleomorphic membrane compartments.** (A) Confocal stack of a Myc–GRAF1- and GFP–Cdc42-Q61L-expressing HeLa cell, presented as a maximum intensity projection and a 90° tilted 3D volume. Scale bar: 10 μm. (B) Correlative microscopy analysis of a GFP–GRAF1 and mCherry–Cdc42-Q61L-expressing HeLa cell. The left panel depicts the epifluorescence and electron micrographs. White arrowheads mark the analysed structures positive for both proteins. Colour-coded 3D models derived from the reconstructed electron tomograms and magnifications of two of the analysed clusters are visualised in the right panel. The white arrow points at a tube connected to the cell surface. Scale bars: 100 nm. (C) Representative examples of the two types of membrane compartments recognised within the analysed clusters in B (disc-like and tube-like) and a clathrin-coated vesicle (CCV). Scale bars: 50 nm. (D) Widest diameter of minor axis measurements from the analysed membrane compartments in B. 136 structures from 11 clusters in one cell were included in the analysis. The mean±s.d. is indicated.

Consistent with the proposed involvement of the GTPase Cdc42 and the GAP GRAF1 in the generation of CLICs, GRAF1 localised with a dominant-active form of Cdc42 (Cdc42-Q61L, which is deficient in GTP hydrolysis) to discrete assemblies in fixed cells (Fig. 1A). As a step towards determining their identity, correlative light and electron tomography microscopy (CLEM) was used to resolve the ultrastructure of these assemblies. High-pressure frozen cells transfected with GFP–GRAF1 and mCherry–Cdc42-Q61L were further prepared for CLEM as described in the Materials and Methods (Kukulski et al., 2011; Nixon et al., 2009). By overlaying the captured epifluorescence micrograph with high-resolution electron tomograms acquired from the same sample and region of interest (ROI), it was apparent that all the fluorescent spots containing both proteins overlapped with small membranous structures (Fig. 1B). Cdc42-Q61L fluorescence alone, commonly seen lining the cell edge, was instead associated with the plasma membrane.

Three-dimensional (3D) models of the membranous structures, reconstructed from the tomograms, revealed that the GRAF1- and Cdc42-Q61L-positive structures detected by epifluorescence microscopy actually corresponded to tightly packed clusters of membrane carriers (Fig. 1B). Most of the carriers were separated from the plasma membrane, but examples of tubular structures connected to the cell surface were also found (Fig. 1B). Although they were quite morphologically diverse, the modelled carriers could roughly be divided into being either disc-like or tube-like on the basis of their shape (Fig. 1C; compare to the GRAF1- and Cdc42-Q61L-negative clathrin-coated vesicle). The measurement of the minor axis of each structure further indicated a surprisingly conserved diameter, spanning 20–60 nm in 97% of the carriers (Fig. 1D). The close proximity of the GRAF1- and Cdc42-Q61L-positive carriers to the basal cell surface, evident using confocal microscopy (Fig. 1A), was further confirmed by the reconstructed 3D models. The carrier clusters were detected within 500 nm of the basal membrane, and their distance to the cell edge, as measured in the *x*-*y* plane, ranged between 360 and 2200 nm. Taken together, the information collected from light and electron microscopy shows that GRAF1 and GTPase-deficient Cdc42 localise to carriers formed near the cell surface that have a morphology consistent with the membrane compartments previously defined as CLICs.

### GRAF1 is a marker of CLICs that are rapidly forming at the leading edge of cells

**Fig. 2.**
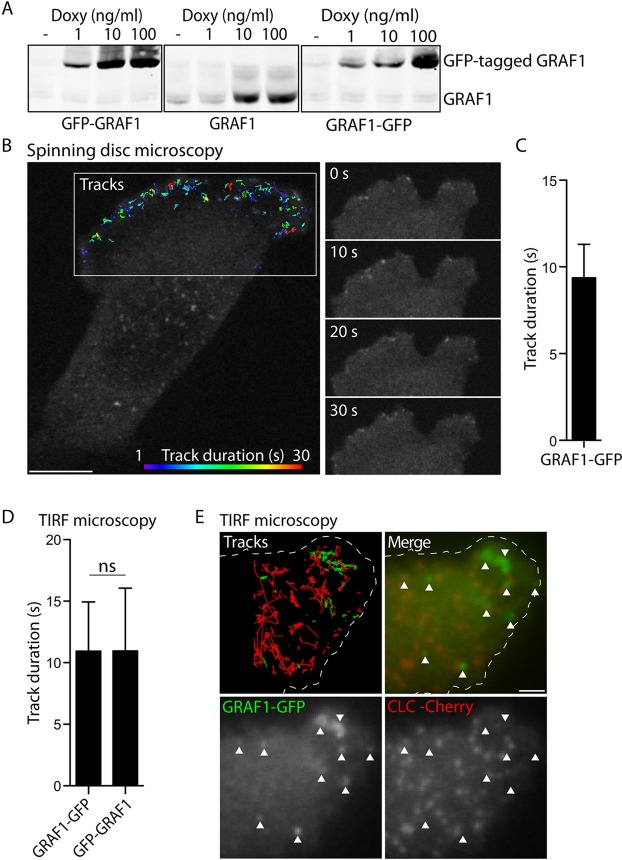
**Dynamic assembly of GRAF1 at the leading edge.** (A) Immunoblots detecting endogenous and recombinant GRAF1 in Flp-In T-REx HeLa GFP–GRAF1, GRAF1 and GRAF1–GFP cell lysates after induction with the stated doxycycline (Doxy) concentrations for 24 h. A doxycycline concentration of 1 ng/ml was chosen for all subsequent experiments. (B) Fluorescence micrograph detecting GRAF1–GFP, corresponding to the last frame of a live-cell spinning disc confocal microscopy acquisition. At the leading edge, tracks of structure movement and duration over time are illustrated as colour-coded lines. Representative frames from the same acquisition are presented as a time series. Scale bar: 10 μm. (C) Duration time of GRAF1–GFP structure tracks derived from acquisitions exemplified in B. 19 cells from three independent experiments were analysed. The bar graph depicts the distribution of the mean±s.d. values derived from each evaluated cell. (D) Duration time of GRAF1–GFP and GFP–GRAF1 structure tracks derived from live-cell TIRF microscopy acquisitions. Bar graphs depict the distribution of the mean±s.d. values derived from each cell included in the respective analysis. Three independent experiments were analysed (Mann–Whitney U test, *n*=11–12 cells, α=0.05, ns, not significant). (E) Fluorescent micrograph showing GRAF1–GFP and mCherry-tagged clathrin light chain (CLC) at a protrusion, corresponding to one frame from a live-cell TIRF microscopy acquisition. Tracks visualise the detected structure movement in the respective channel. The edge of the cell is outlined in the upper panels. Arrowheads highlight GRAF1 assemblies. Scale bar: 2 μm.

To enable the dissection of a potential molecular interplay between Cdc42 and GRAF1 during CLIC formation, a cell model system was established utilising GRAF1 as a pathway marker. In a Flp-In T-REx HeLa cell line, untagged or GFP-tagged GRAF1 was placed under the control of a tet promoter modulated by doxycycline, resulting in an inducible system for the expression of a controlled amount of protein (Fig. 2A). The protein expression level adapted for all subsequent experiments was chosen as to enable the detection of fluorescently tagged GRAF1 by microscopy, inevitably resulting in protein levels above the comparatively low endogenous level in this specific HeLa cell line. However, in contrast to using transient overexpression of GRAF1, the developed Flp-In T-REx cell lines importantly allowed more reproducible and homogenous protein levels within the cultures (Fig. S1A). In fixed cells, confocal microscopy showed that GRAF1, independent of the presence of a fluorescent tag, assembled into punctate and, much less frequently, tubular structures at the cell surface (Fig. S1B). In agreement with previous results detecting endogenous and transiently expressed GRAF1 in HeLa cells (Lundmark et al., 2008), these assemblies colocalised with a fraction of the promiscuous cargo cholera toxin B-subunit (CTxB), as assayed after 2 min internalisation at 37°C (Fig. S2A). Moreover, these assemblies were only on very rare occasions found to overlap with clathrin-associated markers like transferrin (Tfn) and AP-2 (the adaptin for clathrin-mediated endocytosis), or the early endosomal marker EEA1 (Fig. S2A).

Analysis by live-cell spinning disc confocal microscopy revealed the formation of abundant short-lived GRAF1–GFP-positive punctae at the leading edge of cells (Fig. 2B; Movie 1). Software was used to create tracks corresponding to each detected structure, and from these the duration time of each track was derived. The average lifetime (track duration) of the dynamic GRAF1 assemblies was thereby determined to be ∼10 s (Fig. 2C), showing that the protein was transiently assembled at the leading edge. This is in agreement with GRAF1 being associated with CLICs, which are dynamically formed at the cell periphery during cell spreading and migration (Doherty et al., 2011a; Howes et al., 2010a). To determine whether GRAF1 assembled at the plasma membrane, cells were followed by live-cell total internal reflection fluorescence (TIRF) microscopy. Structure tracks were created from the acquisitions and the duration of each track was calculated. The lifetimes of GRAF1–GFP and GFP–GRAF1 assemblies were comparable to that detected by spinning disc confocal microscopy, suggesting that the burst in GRAF1 fluorescence corresponded to activity very near or at the cell surface (Fig. 2D; Fig. S1C). In agreement with this, detecting the surface binding and uptake of fluorescently tagged CTxB by performing live-cell TIRF microscopy revealed the recruitment of GFP–GRAF1 to CTxB clusters and the subsequent disappearance of both markers from the surface, indicative of endocytic carrier formation (Fig. S2B). Importantly, in GRAF1–GFP cells co-expressing mCherry-tagged clathrin light chain, live-cell TIRF microscopy followed by track analysis showed no overlap between the two proteins (Fig. 2E; Movie 2), implying that GRAF1 does not aid the process of clathrin-mediated endocytosis. It should be noted that GRAF1 assemblies were not exclusively observed at the leading edge of cells and that an increased fraction of the assemblies in the centre and rear of cells displayed duration times longer than 30 s (Fig. S1D,E).

### Loss of Cdc42 and actin polymerisation impair GRAF1 carrier processing and the fluid-phase endocytic capacity

**Fig. 3.**
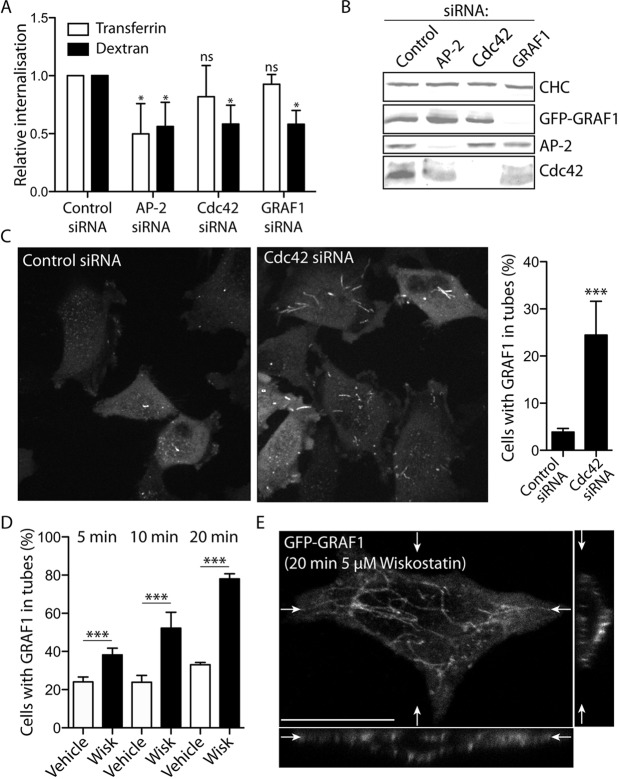
**Inhibition of Cdc42 function and actin polymerisation affects GRAF1 carrier processing.** (A,B) Relative internalisation of aminodextran-S-S-CW800 and transferrin-S-S-CW800 after 30 min uptake in Flp-In T-REx HeLa GFP–GRAF1 cells depleted of AP-2, Cdc42 or GRAF1 by siRNA transfection (A), as confirmed by the immunodetection of the respective protein in comparison to clathrin heavy chain (CHC) in the cell lysates (B). The cargo uptake was normalised to the corresponding control siRNA-transfected sample and the significance in relation to this sample was determined by analyses of at least three independent experiments (mean±s.d.; Mann–Whitney U tests, *n*=3–4 samples, α=0.05; ns, not significant; *P<0.05). (C) Fluorescent micrographs depicting GRAF1–GFP in control cells and cells depleted of Cdc42. The ratio of cells with GRAF1 in at least one tubular structure (i.e. a structure with a length >2 μm) was quantified on the basis of three independent experiments (mean±s.d.; Chi-square test, *n*>450 cells, α=0.05; ***P<0.0001). (D) Ratio of cells with GFP–GRAF1 in tubular structures after treatment with DMSO (Vehicle) or wiskostatin (Wisk), for the indicated time intervals. Cells from three independent experiments were included in the analysis (mean±s.d.; Chi-square tests, *n*>300 cells, α=0.05, ***P<0.0001). (E) Confocal stack of a GFP–GRAF1-expressing cell after 20 min wiskostatin treatment, with presented top and slice views positioned as indicated (white arrows). Scale bar: 10 μm.

Cdc42 was identified as a key regulator of CLIC-mediated endocytosis on the basis of the effects that overexpressed Cdc42 activity mutants exerted on the uptake through this pathway (Sabharanjak et al., 2002). To test the importance of Cdc42 and GRAF1 for clathrin-independent endocytosis in our established Flp-In T-REx HeLa cell lines, fluid-phase internalisation was quantified in cells depleted of either of these two proteins, or of AP-2 by small interfering RNA (siRNA). To control for effects on clathrin-mediated endocytosis, internalisation of transferrin was also assayed. The fluorescence of transferrin-S-S-CW800 or dextran-S-S-CW800 was measured after MesNa-reduction to remove surface-exposed fluorophores, and the internalisation was quantified in relation to that of cells transfected with control siRNA (Fig. 3A,B). Although dextran uptake was impaired in all three samples, transferrin uptake was only significantly reduced in AP-2-depleted cells (Fig. 3A), verifying the importance of Cdc42 and GRAF1 in clathrin-independent endocytosis. The ∼50% reduction of dextran uptake seen in all samples further indicated that clathrin-independent and -dependent pathways, under these conditions, contribute to roughly equal amounts of internalised fluid volume.

To investigate the importance of Cdc42 function on GRAF1-mediated carrier formation, the localisation and behaviour of GRAF1–GFP was followed by live-cell spinning disc confocal microscopy in cells depleted of Cdc42. Although GRAF1 in these cells could still be found in dynamic assemblies at the leading edge, there was a marked increase of GRAF1-decorated tubular structures associated with the cell surface (Fig. 3C; Fig. S3A,B). This implies that GRAF1 in cells with a decreased available pool of Cdc42 can exert its sculpting and/or stabilising function on the plasma membrane, but in the absence of the GTPase that this activity is not properly regulated. Interestingly, active Cdc42 has been shown to be important for recruitment of the Wiskott–Aldrich syndrome protein (WASP)-dependent actin machinery to aid CLIC formation (Chadda et al., 2007). Similar to the effect seen after Cdc42 depletion, cells treated with the WASP-inhibitor wiskostatin showed a time-dependent increase of GFP–GRAF1-decorated tubes associated with the cell surface (Fig. 3D,E). The same phenotype was also observed after treatment with the actin-depolymerising drugs latrunculin A and cytochalasin D (Fig. S3C), previously shown to inhibit uptake through CLICs (Chadda et al., 2007). The altered processing of GRAF1-mediated carriers in Cdc42-depleted cells is likely to be at least a contributing factor to their impaired endocytic capacity, and a result of dysregulated Cdc42-dependent actin dynamics.

### GRAF1 assembly at Cdc42-enriched microdomains antagonises the local plasma membrane association of the GTPase

**Fig. 4.**
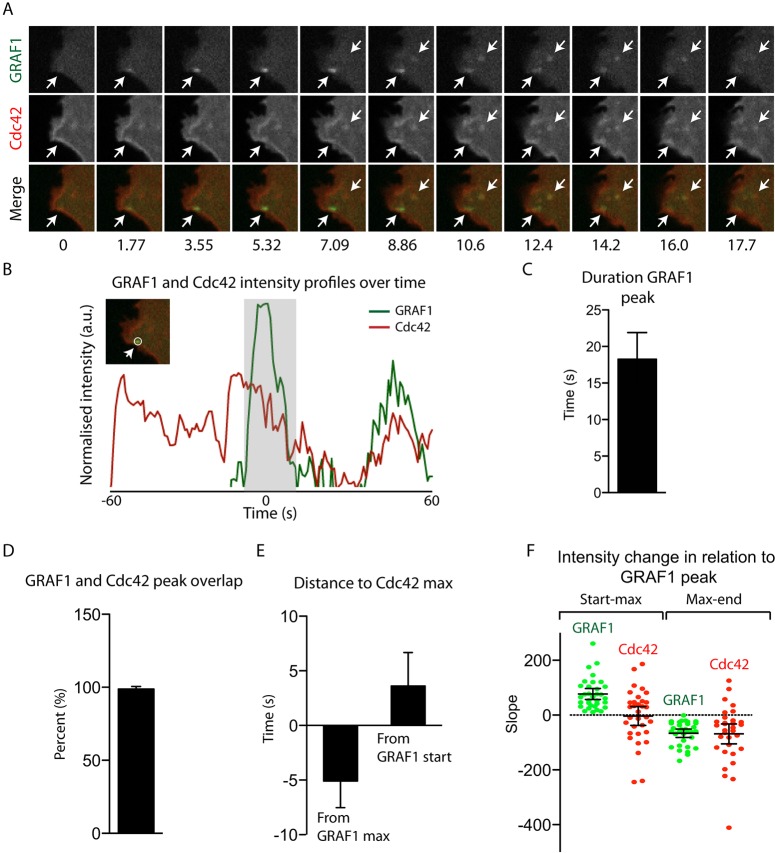
**GRAF1 recruitment to Cdc42 microdomains coincides with a local decreasing plasma membrane association of the GTPase.** (A) Time series from a live-cell spinning disc confocal microscopy acquisition, visualising a protrusion from a Flp-In T-REx HeLa GRAF1–GFP cell co-expressing mCherry–Cdc42. White arrows mark detected GRAF1 assemblies. (B) Intensity profiles of the green and red channels, calculated over time as described in the Materials and Methods, for the first-appearing GRAF1 assembly in A. The grey area highlights the peak duration for the green channel. The insert visualises the ROI used to derive the plotted fluorescence intensity curves. (C–F) Parameters calculated for single GRAF1 assemblies on the basis of the corresponding intensity peak recorded within the intensity profiles derived from live-cell acquisitions, as exemplified in A and B. The time points for the intensity peak start, maximum and end were defined for each structure and channel as described in the Materials and Methods. 34–36 GRAF1 structures from three cells were included in the analysis and results are mean±s.d.

To study the dynamics of the interplay between Cdc42 and GRAF1 during carrier formation, mCherry-tagged wild-type Cdc42 was co-expressed in GRAF1–GFP Flp-In T-REx HeLa cells, and the two proteins followed over time with live-cell spinning disc microscopy (Fig. 4A; Movie 3). Remarkably, 94% of the GRAF1 assemblies that transiently appeared at the leading edge overlapped with membrane-associated Cdc42. Moreover, 88% of these assemblies were recruited to existing microdomains enriched with the GTPase. Further dissection of the indicated interdependency between the two proteins was accomplished by detailed analysis of fluorescence intensity plots created for each detected structure and channel over time (Fig. 4B). The lack of major lateral movements in the detected GRAF1 punctae allowed the intensity value calculations to be restricted to circular ROIs (Fig. 4B). The peak range and maximum was determined for each structure and channel, as described in the Materials and Methods. An average GRAF1 assembly lifetime of 18 s was approximated on the basis of the determined peak ranges, which was slightly longer, but in the same range, as the ∼10 s calculated from the track durations (Fig. 4C, compare to Fig. 2C). Quantification of the lifetime overlap between GRAF1 and Cdc42 assemblies averaged 99%, consistent with the visual assessment above (Fig. 4D). Interestingly, there was a clear trend of Cdc42 reaching the maximum intensity value before GRAF1 (on average 5 s before the GRAF1 intensity maximum, and 3 s after the start of the GRAF1 peak) (Fig. 4E). Consistent with this, for 49% of the analysed structures, it was evident that the Cdc42 intensity decreased as the GRAF1 intensity increased (compared to 82% when analysing the GRAF1 intensity decrease) (Fig. 4F). This suggests that GRAF1 somehow antagonises the existing cell surface enrichment of Cdc42 as it assembles on the membrane, likely by mediating its internalisation and/or transiently interacting with the GTPase to switch it off and thereby destabilising its association with the microdomain.

### An interaction between the GAP domain and transition state Cdc42 enforces membrane assembly of GRAF1

**Fig. 5.**
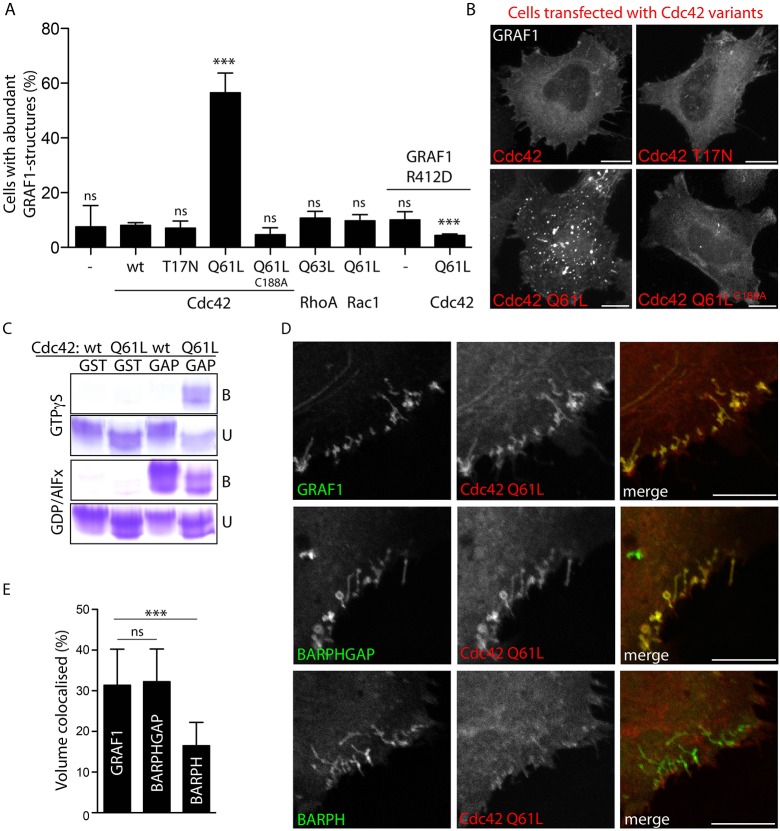
**Interaction with transition state Cdc42 enforces membrane assembly of GRAF1.** (A) Ratio of Flp-In T-REx HeLa cells showing a phenotype of abundant (≥15) GFP–GRAF1 or GFP–GRAF1-R412D structures in the absence (−) or presence of co-expressed *CoralHue*-tagged small GTPases and mutants thereof. For statistical analysis, cells from at least three independent experiments were included. The results were compared to the GRAF1 samples expressing wild-type Cdc42, or in the case of GRAF1-R412D cells, were related to the corresponding GRAF1 samples (mean±s.d.; Chi-square tests, *n*≥300 cells, α=0.05; ns, not significant; ***P<0.0001). (B) Maximum intensity projections of confocal stacks detecting GFP–GRAF1 in cells co-expressing the indicated *CoralHue*-tagged Cdc42 proteins. (C) Coomassie-stained SDS-PAGE gel from a pulldown experiment using purified GST (GST) or a GST-tagged GRAF1 GAP domain (GAP) as bait, and purified Cdc42 or Cdc42-Q61L loaded with GTPγS or GDP in the presence of AlF_x_ (GDP/AlF_x_) as prey. The bound (B) and unbound GTPases (U) were detected. (D) Maximum intensity projections of confocal stacks detecting GFP–GRAF1 or indicated truncates (the BAR-PH-GAP and BAR-PH domains) and *CoralHue*–Cdc42-Q61L in transfected HeLa cells. (E) Percentage volume of GFP–GRAF1 proteins colocalised with *CoralHue*–Cdc42-Q61L, as visualised in D. Three independent experiments were analysed (mean±s.d.; Kruskal-Wallis test, Dunn's post test, *n*=15 cells, α=0.05; ns, not significant; ***P>0.0001). Scale bars: 10 μm.

To investigate the impact of the Cdc42 activity state on GRAF1 membrane assembly, GFP–GRAF1 Flp-In T-REx HeLa cells co-expressing CoralHue-tagged dominant-negative (nucleotide-binding deficient T17N), wild-type and dominant-active Cdc42 were analysed by confocal microscopy. In comparison to the other two samples, Cdc42-Q61L-transfected cells showed a striking accumulation of GRAF1 in bright punctate and tubular structures (Fig. 5A,B), reminiscent of the clustered carriers formed in HeLa cells transiently co-expressing the two proteins (compare with Fig. 1A). Moreover, this phenotype was dependent on the membrane localisation of the GTPase because it was abolished after introducing a point mutation of the cysteine residue (C188A) responsible for anchoring Cdc42 to the membrane (Fig. 5A,B). Notably, no accumulation of GRAF1 structures was seen in cells co-expressing dominant-active RhoA-Q63L or Rac1-Q61L (Fig. 5A), two other GTPases within the same Rho family of actin regulators, confirming the specificity of the induced phenotype.

*In vitro* pulldown experiments were used to determine whether the Cdc42-Q61L mutation somehow influenced the otherwise inherently transient interaction between GRAF1 and Cdc42, to thereby so profoundly affect GRAF1 membrane assembly. Using purified GST-tagged GRAF1 GAP domains as bait and Cdc42 or Cdc42-Q61L loaded with GTPγS (a more stable GTP analogue) as prey, revealed a preference in binding to the mutant (Fig. 5C). However, repeating the assay with GDP-loaded GTPase in the presence of AlF_x_, to mimic the transition state conformation (Mittal et al., 1996), resulted, in addition, to detectable binding to wild-type Cdc42 (Fig. 5C). The Q61L mutation in Cdc42 increased the affinity to GRAF1, likely by imposing an active site conformation, suggesting that the accumulation of GRAF1 assemblies in cells overexpressing this mutant is the effect of an enforced prolonged direct interaction.

To test this hypothesis *in vivo*, HeLa cells transiently co-expressing CoralHue-tagged Cdc42-Q61L and a GFP-tagged GAP domain containing or lacking truncates of GRAF1, were analysed by confocal microscopy. Like full-length GRAF1, the BAR-PH-GAP truncate showed an extensive accumulation in punctate and tubular structures together with the mutated GTPase (Fig. 5D,E). Importantly, this effect was not seen with GFP–BAR-PH, confirming that it was dependent on the presence of the GAP domain. To avoid potential averse consequences of overexpressing truncated proteins, a Flp-In T-REx HeLa cell line was established with an inducible expression of GFP–GRAF1-R412D, a point mutant disrupted in the so-called arginine finger known to be vital for the GAP activity (Jelen et al., 2009) (Fig. S3D,E). Co-expression of Cdc42-Q61L in this cell line did not result in any accumulation of GRAF1-R412D structures (Fig. 5A; Fig. S3G), verifying that the enforced GRAF1 membrane accumulation induced by GTPase-deficient Cdc42 is a consequence of a direct interaction between the two proteins.

### GTP hydrolysis deficiency traps Cdc42 together with GRAF1 on endocytic vesicles

**Fig. 6.**
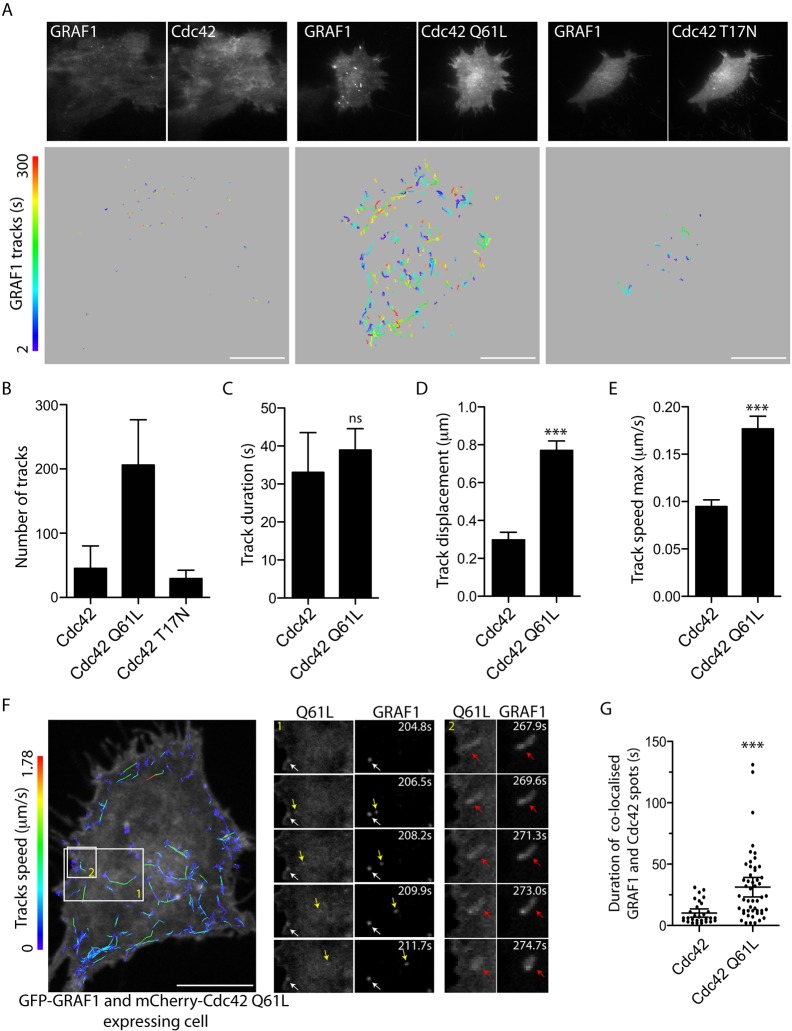
**Cdc42 GTPase deficiency affects GRAF1 carrier dynamics after their formation.** (A) Fluorescence micrographs depicting the start frames from live-cell TIRF microscopy acquisitions of GFP–GRAF1 structures in Flp-In T-REx HeLa cells transfected with the indicated *CoralHue*-tagged Cdc42 constructs (upper panels). The corresponding structure tracks over time, derived from the acquisitions, are represented as colour-coded lines (lower panels). Structures detected over the entire basal cell surface were included in the track analysis. (B–E) Quantification of the number and dynamic parameters of GFP–GRAF1 structure tracks from acquisitions exemplified in A. Three independent experiments were analysed [Student's *t*-tests on log_10_-transformed data, *n*≥136 structure tracks (from four cells), α=0.05; ns, not significant; ***P>0.0001]. Bar graphs in C–E depict the distribution of the mean±s.d. values derived for each cell included in the respective analysis. (F) Fluorescence micrograph showing the first frame from a live-cell spinning disc confocal microscopy acquisition of GFP–GRAF1 structures in cells transfected with the indicated *mCherry-*tagged Cdc42-Q61L. Speeds measured over the detected GRAF1 tracks are represented as colour-coded lines. Magnifications of areas 1 and 2 are visualised as a time series to highlight the mobile membrane structures positive for both proteins. The white arrow marks a structure at the cell surface, the yellow arrow follows the lateral movement of an internal structure and the red arrow points out the fission of a tubular structure from the cell surface. (G) Quantification of duration of colocalisation between GFP–GRAF1 membrane assemblies and respective indicated *mCherry-*tagged Cdc42 protein, based upon track analyses performedon** live-cell spinning disc confocal microscopy acquisitions like that exemplified in F. Cells from two independent experiments were analysed and the mean±s.d. is indicated [Student's *t*-test on log_10_-transformed data, *n*≥29 structure tracks (from three cells), α=0.05; ***P>0.0001). Scale bars: 10 μm.

The confirmed interaction between GRAF1 and Cdc42 *in vivo*, begged the question as to whether GRAF1-mediated inactivation of Cdc42 was important during endocytic carrier formation. To resolve how Cdc42 activity influenced the formation of endocytic carriers, the dynamics of GFP–GRAF1 assemblies in Flp-In T-REx HeLa cells co-expressing CoralHue-tagged wild-type Cdc42, Cdc42-T17N or Cdc42-Q61L were analysed by live-cell TIRF microscopy. Individual GRAF1 structures detected over the whole basal membrane were tracked over time in the acquisitions (Fig. 6A) and used to calculate the number of assemblies, their lifetime (track duration), the distance between their appearance and disappearance (track displacement), and the maximum speed of the structures (Fig. 6B–E). Consistent with the results from fixed cells, co-expression of Cdc42-Q61L resulted in four times more GRAF1 assemblies than detected in the other samples (Fig. 6B). Notably, no clear difference in lifetime (track duration) of GRAF1 assemblies was found between Cdc42- and Cdc42-Q61L-expressing cells (Fig. 6C), showing that the GRAF1 membrane accumulation enforced by Cdc42-Q61L was not trapping the protein at the cell surface. However, the GRAF1 structures in cells co-expressing the GTPase-deficient mutant exhibited both an increased lateral mobility (track displacement) and a higher maximum speed (Fig. 6D,E). Although the directional movement revealed by the track pattern of GRAF1 structures in Cdc42-Q61L co-expressing cells (Fig. 6A) was unlikely to represent diffusion within the membrane, it could be explained by enforced GRAF1 assembly on intracellular vesicles trafficked along cytoskeletal tracks near the cell surface that are in close enough proximity to the plasma membrane to be detected within the TIRF field.

To further explore the mobility of the GRAF1 assemblies enforced by co-expression of GTPase-hydrolysis-deficient Cdc42, live-cell spinning disc microscopy was employed to increase the depth of the fluorescence probe detection in the focal plane of interest (i.e. a plane close to, but just above, the basal membrane). In GFP–GRAF1 cells co-expressing mCherry–Cdc42-Q61L, the proteins localised together both on budding carriers and intracellular laterally mobile vesicles (Fig. 6F; Movie 4). The appearance of the latter type of structures in the TIRF and captured confocal fields indicates that carriers formed from the cell surface, positive for both proteins, later stay or reappear in a plane near the plasma membrane. From live-cell spinning disc microscopy acquisitions of cells co-expressing either wild-type Cdc42 or Cdc42-Q61L, the detected structures of GRAF1 and respective GTPase protein were tracked and the time range of colocalisation was calculated. The colocalisation of GRAF1 and Cdc42 was significantly more transient than that of GRAF1 and Cdc42-Q61L (Fig. 6G), suggesting that the prolonged interaction between GRAF1 and GTP-hydrolysis-deficient Cdc42 results in a longer co-existence of the proteins on internalised vesicles (i.e. an entrapment of both proteins on budded vesicles).

### Inactivation of Cdc42 by GRAF1 is not required for fluid-phase uptake but for the intracellular maturation of CLICs

**Fig. 7.**
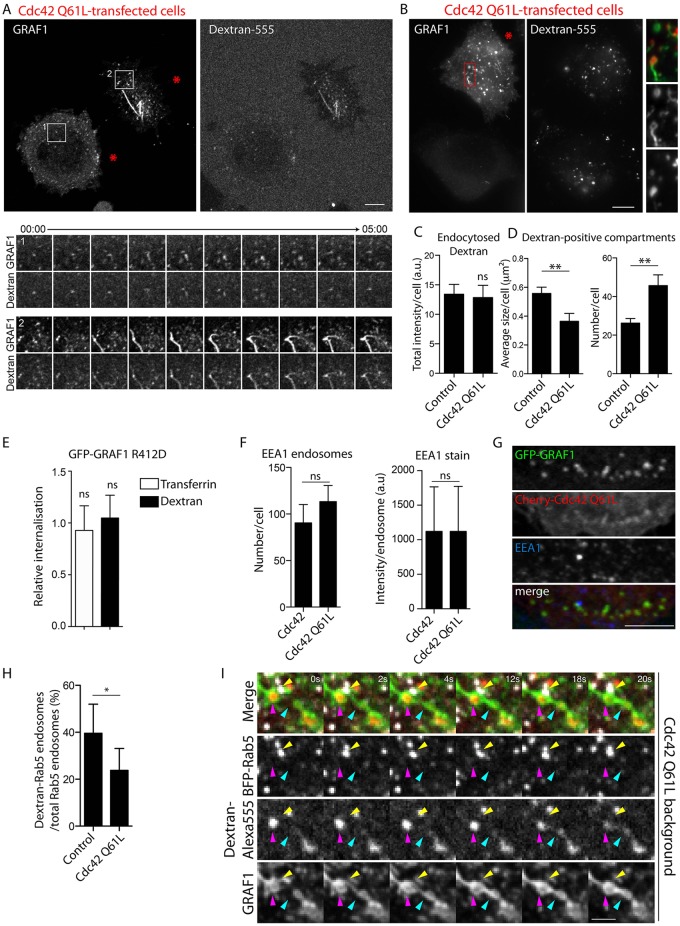
**Cdc42 GTPase deficiency disrupts the maturation of internalised GRAF1 carriers.** (A) Fluorescence micrograph representing the first frame of a live-cell confocal acquisition detecting dextran­–Alexa-Fluor-555 and GFP–GRAF1 in Flp-In T-REx HeLa cells co-expressing Myc–Cdc42-Q61L (upper panels). The acquisition was started after a 5-min incubation with the cargo followed by medium exchange. Analysis on the basis of four captured cells revealed dextran enrichment in 31±16% (mean±s.e.m.) of the identified GRAF1 structures. Areas 1 and 2 from the acquisition are presented as time series (lower panels). Red stars indicate cells affected by Myc–Cdc42-Q61L. Scale bar: 10 μm. (B) Epifluorescence micrograph of GFP–GRAF1 and dextran structures after a 5-min uptake in two cells, one defined as phenotypically unaffected (control) and the other defined as affected by Myc–Cdc42-Q61L (red star). Scale bar: 10 μm. (C,D) Quantification of the total dextran uptake and descriptive parameters for dextran-positive compartments for comparison of GFP–GRAF1 cells unaffected and affected by the Cdc42-Q61L transfection, as defined in B, to circumvent inter-sample variation of cargo fluorescence. Error bars represent the s.e.m. from three independent experiments (Mann–Whitney U tests, *n*=12–14 cells, α=0.05; ns, not significant; **P<0.01). (E) Relative internalisation of aminodextran-S-S-CW800 and transferrin-S-S-CW800 after a 30-min uptake in Flp-In T-REx HeLa GFP–GRAF1-R412D cells. The cargo uptake of the mutant-expressing cells collected from at least three independent experiments was normalised to the corresponding wild-type sample and the significance in relation to this sample was assessed (mean±s.d.; Mann–Whitney U tests, *n*=3–4, α=0.05; ns, not significant). (F) EEA1 intensity per endosome and number of EEA1-positive endosomes per GFP–GRAF1 cell transfected with mCherry-tagged Cdc42 or Cdc42-Q61L. Three independent experiments were analysed [mean±s.d.; left diagram, Student's *t*-test, *n*>13,500 EEA1-stained endosomes (from 50 cells), α=0.05; ns, not significant; right diagram, Mann–Whitney U test, *n*=3 cells, α=0.05; ns, not significant). Bars in the left graph depict the distribution of the mean values derived from each experiment. (G) Confocal fluorescence micrographs of GFP–GRAF1 cells transfected with mCherry–Cdc42-Q61L and immunostained with anti-EEA1 antibody. Scale bar: 5 μm. (H) Ratio of dextran-filled Rab5 compartments in BFP–Rab5-transfected GFP-GRAF1 cells without or with co-expressed Myc–Cdc42-Q61L after a 5-min cargo uptake. Cells from three independent experiments were included in the analysis [mean±s.d.; Mann–Whitney U test, *n*≥5 captured frames (at least 5 cells), α=0.05; *P<0.05]. (I) Representative time series from a live-cell spinning disc confocal microscopy acquisition visualising the dynamics of dextran-filled internal compartments in GFP–GRAF1 cells co-expressing BFP–Rab5 and Myc–Cdc42-Q61L. The yellow arrow marks a Rab5 vesicle devoid of dextran, adjacent to a cargo-filled GRAF1 compartment pointed out by the magenta arrow. The turquoise arrow marks a second GRAF1-labelled structure devoid of dextran, which over the depicted time range fuses with the first GRAF1 compartment (specifically note the redistribution of cargo between these two compartments). Scale bar: 2 μm.

The potential effects on endocytosis inferred by overexpression of Cdc42-Q61L were further assayed by analysing the uptake of dextran. Dextran–Alexa-Fluor-555 was added to GFP–GRAF1 Flp-In T-REx HeLa cells co-expressing Myc–Cdc42-Q61L as a 5-min pulse, then a quick wash was used to remove the excess extracellular marker and probes were followed by live-cell confocal microscopy. Immediately after the pulse, dextran was enriched in approximately one third of the accumulated GRAF1 structures (Fig. 7A), verifying that these correspond to plasma-membrane-derived and budded endocytic carriers. The same protocol was used to prepare fixed samples for epifluorecence microscopy. Dextran could also be visualised in GRAF1-positive carriers under fixed conditions (Fig. 7B), but it should be noted that permeabilisation of fixed samples was avoided, given that the detection of this marker deteriorates with detergent treatment. Using software to define dextran compartments from the captured images, it was determined that the total uptake of fluid-phase was not significantly altered in cells affected by the Cdc42-Q61L transfection (Fig. 7C). Also in line with this, GFP–GRAF1-R412D cells showed a higher prevalence of membrane assemblies in comparison to GFP–GRAF1 cells (Fig. S3H), without any detectable effect on dextran endocytosis (Fig. 7E; Fig. S3F), further verifying that the GRAF1 GAP activity against Cdc42 was not required for endocytic carrier formation.

The entrapment of GRAF1 and Cdc42-Q61L on erratically behaving internalised vesicles, pointed towards a post-fission trafficking defect. Strikingly, in GRAF1 and Cdc42-Q61L co-expressing cells with accumulated GRAF1 assemblies, significantly more, but smaller, dextran-positive compartments were detected (Fig. 7D). This indicated that these internal carriers were stalled in their endosomal maturation or that early endosomes were fragmented under these conditions. However, there was no significant effect on the number of early endosomes or the total intensity of the early endosomal marker EEA1 (Fig. 7F). Furthermore, carriers decorated by GRAF1 and Cdc42-Q61L were never positive for EEA1 (Fig. 7G), suggesting that these more likely represented a trapped intermediate membrane compartment. To explore this in more detail, dextran trafficking was captured in GFP–GRAF1 cells co-expressing BFP–Rab5 alone, or together with Myc–Cdc42-Q61L, by live-cell spinning disc microscopy. Cells were pulsed with the cargo for 5 min, quickly washed and the dynamics of the probes were monitored over 5 min. At the start of the acquisition, there was a significantly lower ratio of dextran-filled Rab5 vesicles in cells affected by the co-expression of GTPase-deficient Cdc42 (Fig. 7H; Fig. S4A), showing that trafficking of internalised dextran to this early endosomal compartment was negatively affected. It should also be noted that there was a high number of dextran-containing vesicles that, at the end of the acquisition, were still devoid of Rab5, even in cells not co-expressing Cdc42-Q61L, likely corresponding to alternative trafficking routes. Although fusion events could be distinguished between dextran-filled Rab5 compartments (Fig. S4B), and between separate GRAF1 compartments in cells co-expressing Cdc42-Q61L (Fig. 7I), no example of fusion between GRAF1 and Rab5 compartments was found despite frequent transient engagements observed between trapped GRAF1 compartments and labelled early endosomes (Fig. 7I). As reflected by the Cdc42-Q61L-enforced clustering of GRAF1 carriers resolved by CLEM, taken together, our study shows that GRAF1-mediated inactivation of Cdc42 is not vital for the formation and budding of endocytic carriers, but for their further intracellular maturation.

## DISCUSSION

Cell spreading and migration involve dynamic alterations in cell surface turnover supporting local membrane rigidity and flexibility through directed trafficking of lipids and surface receptors. This is reflected in the polarised activities of endocytic pathways including caveolae concentrated at the lagging edge, and fast endophilin-mediated endocytosis (FEME) and CLICs enriched at the leading edge (Boucrot et al., 2015; Chaudhary et al., 2014). Here, we show that direct interplay between GRAF1 and Cdc42 coordinates maturation of CLICs at the cell front and hence constitutes an efficient machinery for aiding in the turnover of membrane constituents. We found that GRAF1 dynamically assembled at the plasma membrane in discrete areas that did not overlap with the formation of clathrin-coated pits. The assemblies of GRAF1 were short lived and preceded by local membrane enrichment of Cdc42, suggesting that GRAF1 is transiently recruited to membrane microdomains imposed by upstream Cdc42 activity. The concentration of GRAF1 will increase the local curvature of the membrane through the BAR domain, promoting membrane invagination. At the same time, this results in high spatially confined GAP activity to stimulate GTP hydrolysis by Cdc42. The inactivation of Cdc42 has been shown to reduce the membrane domain association and thereby results in a more dynamic pool of Cdc42 (Bendezú et al., 2015; Chadda et al., 2007). Indeed, the progressive assembly of GRAF1 correlated with a decrease in local Cdc42 enrichment. Given that we could not detect Cdc42 on internal carriers together with GRAF1, we suggest that the transient interaction between the two proteins results in inactivation and a subsequent decrease in the membrane association of Cdc42. This spatial restriction of Cdc42 activity could promote the identity transition from plasma membrane to an internal membrane compartment and facilitate further maturation. Based on this, we propose that the key role of GRAF1 in regulating turnover at the leading edge explains the previously described defect in the fusion of myoblasts and impaired migratory behaviour of cells lacking GRAF1 (Doherty et al., 2011a,b; Lenhart et al., 2014).

The initiation of the budding process and activation of Cdc42 might be facilitated by GEFs in response to extracellular signals, cellular polarisation or alterations in membrane tension. There are likely to be additional factors that are involved in the initiation and progression of CLIC formation. Previously, Arf1 has been shown to be essential for CLIC uptake, and the activation of Arf1 at the cell surface has been proposed to recruit ARHGAP10 to modulate Cdc42 activity (Kumari and Mayor, 2008). It is not yet known whether GRAF1 is directly coupled to these components or whether they regulate similar or distinct subtypes of CLICs. Actin polymerisation appears to play a central role in CLIC formation. Local actin-driven organisation of the cell surface has been suggested to promote endocytosis through clustering of lipids and proteins (Gowrishankar et al., 2012; Romer et al., 2010). In addition, actin polymerisation has been proposed to facilitate the release of carriers from the surface in a process controlled by Cdc42 (Chadda et al., 2007). Interestingly, we found that Cdc42 and the downstream WASP-mediated actin polymerisation were vital for processing of GRAF1-positive carriers. Depletion of Cdc42 or the acute inhibition of actin polymerisation resulted in extended GRAF1-decorated tubules associated with the cell surface. This shows that Cdc42 is not essential for GRAF1 membrane assembly, but suggests that Cdc42-driven actin polymerisation plays a central role for processing of membrane invaginations generated by this mechanism. The effect on carriers could be due to impaired scission from the cell surface or reduced support from local cortical actin that relieves membrane tension and thereby promotes tubulation of the cell surface. Interestingly, it has recently been shown that perturbation of the cortical actin by inhibition or depletion of Cdc42 results in reduced membrane tension (Bretou et al., 2014).

We show that the depletion of either Cdc42 or GRAF1 results in a striking reduction in fluid uptake, consistent with that the cycling activities of Cdc42 substantially regulate the dynamics and turnover of the plasma membrane (Kumari et al., 2010). However, the precise influence and effects of GTP-binding and hydrolysis has so far not been understood. Here, we show that the inactivation of Cdc42 was not required for the generation or the internalisation of GRAF1-positive CLICs given that neither the duration time of GRAF1 assemblies at the surface nor the total amount of internalised fluid was influenced by expression of Cdc42-Q61L or GRAF1 mutated in the GAP domain. However, we show that inability of Cdc42 to hydrolyse GTP results in the accumulation of rapidly moving internal carriers coated by both GRAF1 and Cdc42. Analysis of these carriers by CLEM showed that they were composed of multiple small vesicular and tubular structures with a mean diameter of 50 nm. This is in agreement with the previously described morphology of CLICs, and the proposed membrane curvature promoted by the BAR domain of GRAF1 (Howes et al., 2010a; Lundmark et al., 2008). We show that the Q61L mutation increases the affinity of Cdc42 for the GAP domain of GRAF1, which could explain the prolonged time of residence of GRAF1 and Cdc42 on the carrier membrane. Our results suggest that this affects the maturation of carriers, possibly due to altered recruitment of endosomal proteins and lipid turnover. We found that the stalled carriers were able to fuse with each other but not with Rab5-positive endosomes, suggesting that they were trapped in their membrane identity. CLIC and GEEC carriers have been shown to undergo homotypic and endosomal fusion (Kalia et al., 2006). We did not detect GRAF1 in EEA1-positive endosomes, suggesting that this protein likely comes off the nascent vesicle to allow fusion with endosomal compartments. Taken together, the transient interaction between GRAF1 and Cdc42 will terminate Cdc42 activity and promote transition of the local PIP_2_-enriched membrane environment to enable release of GRAF1 and promote endosomal maturation. This might be facilitated by the phosphoinositide 3-kinase-driven conversion of the lipid environment. Indeed GRAF1, but not Cdc42, has been detected on CLICs stalled by wortmannin treatment, which inhibits phosphoinositide 3-kinase (Howes et al., 2010a).

We have previously shown that GRAF1 is detected in pleomorphic structures (Lundmark et al., 2008). Because there is no definite cargo known for CLICs, it is difficult to know whether these structures represent distinct compartments or different steps of maturation of carriers. Characterisation of GRAF1-positive carriers using the Flp-In T-Rex HeLa cell system revealed that the majority of GRAF1 structures detected at the cell periphery were small and transient. We also observed longer tubes, likely corresponding to the previously described endogenous GRAF1-containing tubes (Lundmark et al., 2008), suggesting that GRAF1 is involved in further membrane maturation events under some conditions. The stabilisation of CLIC and GEEC intermediates upon the expression of Cdc42-Q61L phenotypically resembles the previously described trapping of an endosomal compartment induced by constant Arf6 activity (Naslavsky et al., 2004). Interestingly, the analogous Arf GAPs of the ASAP family are involved in regulating cytoskeletal and membrane reorganisation (Randazzo et al., 2007), and might influence Arf activity and membrane curvature through a similar co-operative regulatory mechanism as proposed here. We believe that these types of systems should not be considered canonical means for receptor sorting and vesicle generation. Instead, they are optimised to regulate cell surface dynamics and the recycling of membrane reservoirs in response to external cues such as changes in adhesion or membrane tension.

## MATERIALS AND METHODS

### Antibodies, probes and plasmids

The polyclonal anti-GRAF1 rabbit antibody RA-83 was produced as previously described (Lundmark et al., 2008). Commercially acquired antibodies included rabbit anti-Myc (2272S, Cell Signaling Technology), mouse anti-clathrin heavy chain (clone 23, 610499, BD Transduction Laboratories), mouse anti-AP50 (clone 31, 611351, BD Transduction Laboratories; an AP-2 subunit), rabbit anti-Cdc42 (ab109553, Abcam), mouse anti-EEA1 (610456, Clone 14, BD Transduction Laboratories), mouse anti-GFP (JL-8, 632381, Living Colours) and goat anti-aldolase (AB1809, Chemicon International, Inc.) antibodies. Secondary antibodies conjugated to Alexa Fluor molecules (Molecular Probes), horseradish peroxidase (HRP; Sigma-Aldrich and Agrisera) and IRDye 800CW or 680RD (LI-COR Biosciences) were used for immunofluorescence and western blot detections. The fluorescent DNA probe DRAQ5 was obtained from BioStatus Limited, and the 10 kDa dextran–Alexa-Fluor-555 and CTxB–Alexa-Fluor-647 were purchased from Molecular Probes. Cy3–transferrin, 800CW-S-S-transferrin and 800CW-S-S-aminodextran were produced by covalently coupling respective cargo (Sigma-Aldrich) to amine-reactive Cy3 (Amersham) and 800CW-S-S (LI-COR Biosciences) according to the manufacturer's recommendations. The plasmids used in this study are summarised in Table S1. Constructs were created by PCR amplification of inserts with flanking restriction sites for ligation into corresponding vectors. Amino acid mutations were generated through site-directed PCR mutagenesis with PCR primers specified in Table S2.

### Protein purification and pulldown experiments

GST fusions of wild-type GRAF1, its arginine finger mutated GAP truncate and Cdc42 proteins were purified as previously described (Doherty et al., 2011a). Thrombin (GE Healthcare Life Sciences) was used for tag removal. GST–GAP and GAP-R412D fusions were purified in 25 mM HEPES pH 7.4 and 150 mM NaCl, and Cdc42 proteins in the same buffer supplemented with 5 mM MgCl_2_. The nucleotide loading of GTPases was performed at 30°C for 20 min in the presence of EDTA (twofold molar excess to MgCl_2_) and nucleotide (18-fold molar excess to protein) (GTPγS and GDP from Sigma-Aldrich). MgCl_2_ (twofold molar excess to EDTA) was added to stop the reaction and the experimental buffer conditions were reached by buffer exchange in Micro Bio-Spin P-6 Gel columns (Bio-Rad). For the *in vitro* protein interaction experiments, glutathione–Sepharose-4B bead-bound GST-tagged protein baits were incubated with prey proteins for 1–3 h at 4°C under rotation. The unbound protein was collected by centrifugation (400 ***g*** for 2 min), the bound protein was washed, and both fractions were boiled in sample buffer for analysis by SDS-PAGE. All assays including GDP-loaded GTPase were performed in the presence of AlF_x_ (1 mM AlCl_3_, and 25 mM NaF).

### Cell culture and transfection

HeLa cells (ATCC-CRM-CCL-2) were cultured in Dulbecco's modified Eagle's medium (DMEM; low glucose, L-glutamine, sodium pyruvate, HEPES and Phenol Red), supplemented with 10% fetal bovine serum (FBS; Gibco). Flp-In T-REx HeLa cell lines with tetracycline-inducible expression of GRAF1, GRAF1–GFP, GFP–GRAF1 and GFP–GRAF1-R412D were generated as previously described (Mohan et al., 2015). Established cultures were grown in the mentioned DMEM, exceptions being during 800CW-S-S-cargo internalisation assays and live-cell acquisitions when basic medium containing 4.5 g/l glucose was used (Gibco). The culturing media were further supplemented with 100 μg/ml hygromycin B and 5 μg/ml blasticidin S HCl (Gibco) for plasmid selection, and recombinant protein expression was induced by incubation in doxycycline hyclate (Sigma-Aldrich) for 20±4 h. Targeted protein silencing was accomplished with siRNA against GRAF1 (‘siRNAb’ Stealth RNAi, Invitrogen) (Lundmark et al., 2008), Cdc42 #1 (J-005057-05-0020 ON-TARGET*plus siRNA*, Dharmacon), Cdc42 #2 (VHS40393, Stealth siRNA, Life Technologies) or AP-2 (AP2M1HSS101955 Stealth RNAi, Invitrogen), and a Medium GC Duplex RNA (Stealth RNAi, Invitrogen) served as the negative control. Transfections for transient expression of plasmids (16±4 h) and siRNA (60–96 h) were performed using Lipofectamine 2000 (Invitrogen) according to the manufacturer's recommendations. Protein expression levels were analysed in cleared cell lysates by western blotting and HRP or IRDye antigen detection using a Medical X-ray Processor (Kodak) or an Odyssey Sa reader (LI-COR Biosciences), respectively.

### Cellular uptake and drug treatments

To capture cargo uptake after a given time interval, cells were incubated at 37°C with fluorescently labelled CTxB (3 μg/ml), transferrin (2 μg/ml) or dextran (2 mg/ml) and washed in preheated medium before fixation. For the quantification of internalised IRDye-S-S-linked cargo, cells seeded in 24-well plates were incubated for 30 min at 37°C with 8 μg/ml 800CW-S-S-transferrin or 0.18 mg/ml 800CW-S-S-aminodextran and washed in preheated medium before fixation. At room temperature, the samples were washed in stripping buffer (50 mM Tris-HCl pH 8.7, 100 mM NaCl, and 2.5 mM CaCl_2_), reduced for 1.5 h in the same buffer supplemented with 60 mM MesNa and further washed in phosphate-buffered saline (PBS). Cells were finally stained with 1 μM DRAQ5 for 30 min and washed in PBS before recording probe fluorescence using the Odyssey Sa reader. The uptake was defined as the ratio between the cell number (the measured DRAQ5 fluorescence) and the amount of internalised cargo (the measured 800CW fluorescence). Cargo trafficking was also monitored by live-cell fluorescent microscopy directly after the addition of 3 μg/ml CTxB–Alexa-Fluor-647 or after 5 min of incubation with 0.5–2 mg/ml dextran–Alexa-Fluor-555 followed by a wash with preheated medium. The effects of actin polymerisation drugs on GRAF1 structures were analysed by fluorescence microscopy of fixed cells after treatment with 2 μM cytochalasin D, 2 μM latrunculin A (Sigma-Aldrich), 5 μM wiskostatin (Calbiochem) or DMSO.

### Fluorescence microscopy

Cells were fixed and prepared for immunofluorescence analysis as previously described (Lundmark et al., 2008). Images of fixed cell samples were captured using an epifluorescence Axioimager Z1 system (AxioCam MRm camera) (Zeiss) with the ZEN Software and a 63× lens (Plan-Apochromat 1.40 Oil DIC 0.17) or an A1 R Laser Scanning Confocal Microscope system (ANDOR iXon EMCCD camera) (Nikon Instruments) under control of the NIS-Elements Microscope Imaging Software and a 60× lens (Apochromat 1.40 Oil λS 0.17 WD 0.14, Nikon), at the appropriate excitation and emission wavelengths. Live-cell confocal movies were recorded using the 63× lens in the Nikon system or the 63× lens (Plan-Apochromat 1.40 Oil DIC M27) in a Cell Observer Spinning Disc Confocal Microscope system (ANDOR iXon Ultra) (Zeiss) controlled by the ZEN Software. Real-time TIRF acquisitions were captured by employing the 100× lens in respective system [Apochromat 1.49 Oil 0.13-0.20 DIC N2 (Nikon) or Plan-Apochromat 1.46 Oil DIC M27 (Zeiss)]. Micrographs and the acquired movies were prepared (cropped, rotated, linearly adjusted for intensity and converted) using Adobe Photoshop or ImageJ.

### Correlative microscopy

For correlative fluorescence and electron microscopy, cells were high-pressure frozen on sapphire discs (0.7% low-melt agarose and DMEM; HPM100) and processed by freeze substitution. 250-µm sections were placed on 200 mesh copper grids with a carbon support film (TAAB) and immediately imaged by epifluorescence microscopy, with all above steps performed according to Kukulski et al. (2011, 2012). Grids were further dried, coated with 15-nm gold particles conjugated to protein A (BioCell) and post-stained with 2% uranyl acetate and Reynolds lead citrate. Grids were placed in a high-tilt dual-axis holder (Fischione Instruments) and electron tomograms corresponding to the fluorescent structures of interest (determined from recorded grid positions) were acquired using a Tecnai F30 electron microscope (FEI) at 300 kV, equipped with an Eagel 4 k CCD camera (FEI) using serial electron microscopy (Mastronarde, 2005). The 9400× magnification tomograms were acquired as single-axis and the 15,500× magnification tomograms as dual-axis tilt series over −60° to 60° (1.5° increment). All tomograms were reconstructed, modelled and quantified with the IMOD package version 4.7.10 (Kremer et al., 1996). The 15,500× magnification tomograms used for the detailed models have a voxel size of 7.67 Å. Fluorescent micrographs, models and tomograms were overlaid using Adobe Photoshop.

### Image analysis and quantifications

Protein bands on western blots detected with HRP-conjugated antibodies were determined from scanned images in ImageJ to derive the relative sample protein content. Estimations of the fraction of fixed cells with GFP–GRAF1 proteins in abundant or tubular structures were performed by epifluorescent visual assessment. The Imaris Software V7.5 (Bitplane) was utilised to analyse micrographs captured from fixed and live-cell samples as specified in Table S3. The recruitment of GRAF1–GFP to mCherry–Cdc42 membrane domains was analysed by comparing the fluorescence intensity profiles of each channel over time, from the live-cell confocal spinning disc acquisitions. The mean intensity value within a five-unit-diameter circular ROI, spanning each structure of interest, was obtained for each channel and frame using the ‘Plot Z-axis profile’ tool in ImageJ. The start and end of each intensity peak were defined as the time points where the calculated mean intensity values rose above and declined below, respectively, the background. Parameters of GFP–GRAF1 and mCherry–Cdc42-Q61L carriers calculated from the correlative microscopy were derived from measurements on the constructed 3D image using the IMOD package.

### Statistics

Statistical tests were performed using Prism 5 (GraphPad Software) with the indicated sample size and number of independent experiments. All quantifications are visualised as the mean±s.d. unless otherwise stated.
